# Docetaxel-Resistance in Prostate Cancer: Evaluating Associated Phenotypic Changes and Potential for Resistance Transfer via Exosomes

**DOI:** 10.1371/journal.pone.0050999

**Published:** 2012-12-10

**Authors:** Claire Corcoran, Sweta Rani, Keith O’Brien, Amanda O’Neill, Maria Prencipe, Rizwan Sheikh, Glenn Webb, Ray McDermott, William Watson, John Crown, Lorraine O’Driscoll

**Affiliations:** 1 School of Pharmacy & Pharmaceutical Sciences & Trinity Biomedical Sciences Institute, Trinity College Dublin, Dublin, Ireland; 2 UCD School of Medicine and Medical Science, University College Dublin, Dublin, Ireland; 3 All-Ireland Cooperative Oncology Research Group (ICORG), Dublin, Ireland; 4 ICORG & Adelaide and Meath Hospital incorporating The National Children’s Hospital (AMNCH), Tallaght, Dublin, Ireland; 5 ICORG & Molecular Therapeutics for Cancer Ireland (MTCI), Dublin, Ireland; University of Kentucky College of Medicine, United States of America

## Abstract

**Background:**

Hormone-refractory prostate cancer remains hindered by inevitable progression of resistance to first-line treatment with docetaxel. Recent studies suggest that phenotypic changes associated with cancer may be transferred from cell-to-cell *via* microvesicles/exosomes. Here we aimed to investigate phenotypic changes associated with docetaxel-resistance in order to help determine the complexity of this problem and to assess the relevance of secreted exosomes in prostate cancer.

**Methodology/Principal Findings:**

Docetaxel-resistant variants of DU145 and 22Rv1 were established and characterised in terms of cross-resistance, morphology, proliferation, motility, invasion, *anoikis*, colony formation, exosomes secretion their and functional relevance. Preliminary analysis of exosomes from relevant serum specimens was also performed. Acquired docetaxel-resistance conferred cross-resistance to doxorubicin and induced alterations in motility, invasion, proliferation and anchorage-independent growth. Exosomes expelled from DU145 and 22Rv1 docetaxel-resistant variants (DU145RD and 22Rv1RD) conferred docetaxel-resistance to DU145, 22Rv1 and LNCap cells, which may be partly due to exosomal MDR-1/P-gp transfer. Exosomes from prostate cancer patients’ sera induced increased cell proliferation and invasion, compared to exosomes from age-matched controls. Furthermore, exosomes from sera of patients undergoing a course of docetaxel treatment compared to matched exosomes from the same patients prior to commencing docetaxel treatment, when applied to both DU145 and 22Rv1 cells, showed a correlation between cellular response to docetaxel and patients’ response to treatment with docetaxel.

**Conclusions/Significance:**

Our studies indicate the complex and multifaceted nature of docetaxel-resistance in prostate cancer. Furthermore, our *in vitro* observations and preliminary clinical studies indicate that exosomes may play an important role in prostate cancer, in cell-cell communication, and thus may offer potential as vehicles containing predictive biomarkers and new therapeutic targets.

## Introduction

While docetaxel offers improvement in overall survival for patients with hormone-refractory prostate cancer (HRPC) as evident from two Phase III trials (TAX 327 and SWOG 9916) and subsequent clinical management, unfortunately relapse is almost inevitable. Reasons for failure of docetaxel to increase survival beyond ∼2.5 months have yet to be completely elucidated. Drug resistance is frequently attributed with the over-expression of transporter proteins, including P-glycoprotein (MDR-1/P-gp), associated with the efflux of many anti-cancer (and other) agents [1]–[3]. Furthermore, chemo-resistance has also been shown to contribute to alterations in the invasive and motile phenotype of cells [4]–[7]. Other phenotypic characteristics that may be associated with this problem in prostate cancer have yet to be defined.

Added to this, recent studies suggest that phenotypic changes associated with cancer may be transferred from cell-to-cell *via* microvesicles/exosomes. Exosomes have been described as nano-sized membrane-bound vesicles of endocytic origin [8]. Depending on their cell of origin, these small vesicles have been implicated with several different roles some of which include their association with diseased states such as cancer. Intercellular communication is one such role, through their ability to promote signal transduction [9] and the transfer of membrane receptors, proteins, mRNA, and miRNAs [10], [11] from one cell to another. The relevance of exosomes in terms of their potential to assist in prostate cancer progression and the development of chemo-resistance has yet to be determined.

Due to the complex nature of prostate cancer progression, docetaxel-resistant prostate cell lines were developed and characterised to represent both primary and metastatic tumours as well as androgen-sensitivity and androgen-resistance in prostate cancer. In brief, acquired-resistance to docetaxel, in two prostate cancer cell lines, conferred cross-resistance to the anthracycline, doxorubicin and induced alterations in motility, migration, invasion, proliferation and anchorage-independent growth. Application of exosomes, isolated from docetaxel-resistant DU145RD and 22Rv1RD cancer cells, to docetaxel-sensitive DU145, 22Rv1 and LNCap parent cells conferred a significant increase in resistance to docetaxel to each of these recipient cells. This may, at least in part, be due to transfer of MDR-1/P-gp by exosomes. Exosomes from prostate cancer serum specimens induced a significant increase in cell proliferation and invasion compared to exosomes from age-matched healthy controls. Furthermore, exosomes from sera of patients undergoing a course of docetaxel treatment compared to matched exosomes from the same patients prior to commencing docetaxel treatment, when applied to both DU145 and 22Rv1 cells, showed a correlation between cellular response to docetaxel and patients’ response to docetaxel treatment. These preliminary translational studies further support the clinical relevance of exosomes in prostate cancer.

## Results

### Determining the Extent of Resistance to Docetaxel

As detailed in Table 1, DU145RD and 22Rv1RD cells were found to be approximately 108-, and 71-fold resistant to docetaxel compared to their respective aged-parent cell lines.

**Table 1:** Average IC_50_ values and fold changes of prostate cancer lines and docetaxel-resistant variants.

Anti CancerAgent	DU145	DU145RD		Fold Change	22Rv1	22Rv1RD		Fold Change
**Docetaxel (nM)**	1.7		183.3	108.7±7.4	4		277	71.3±8.4
**Doxorubicin (nM)**	24		97	4.3±1.0	60		500	8.3±2.0
**Fluorouracil (nM)**	70		77	1.2±0.1	43		70	1.6±0.2
**Carboplatin (nM)**	700		700	1.0±0.1	950		2000	2.1±0.6

### Docetaxel-resistant Cells Demonstrate Some Cross Resistance to Other Anti-cancer Agents

Both resistant cell line variants exhibited cross-resistance (4–8 fold) to Doxorubicin (Table 1), while no significant differences in sensitivity to 5-Fluorouracil or Carboplatin were observed for DU145RD or 22Rv1RD in comparison to their respective parent cells.

### Docetaxel-resistant Variants do not Differ in Morphology but have Different Proliferation, Motility and Invasion Phenotypes Versus Parent Cell Lines

**Figure 1:**
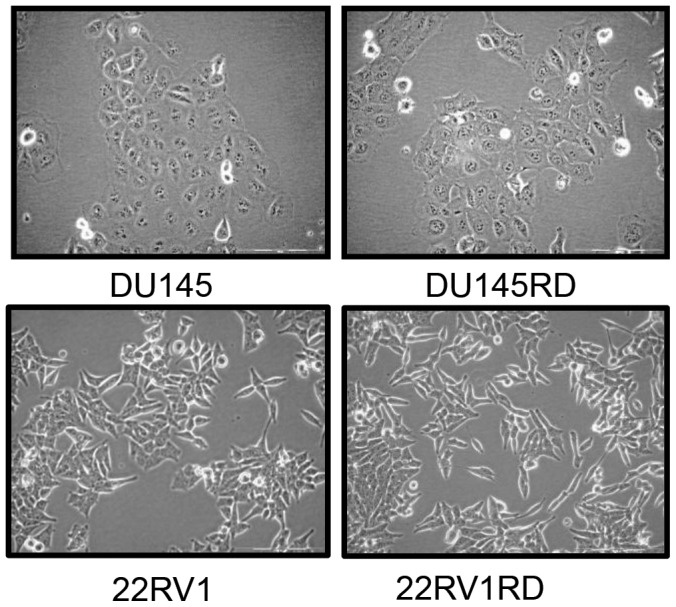
Cell Morphology. Images of sensitive parent and docetaxel-resistant variants of DU145 and 22Rv1 cell lines. (Olympus CKX4, 20X magnification).

**Figure 2:**
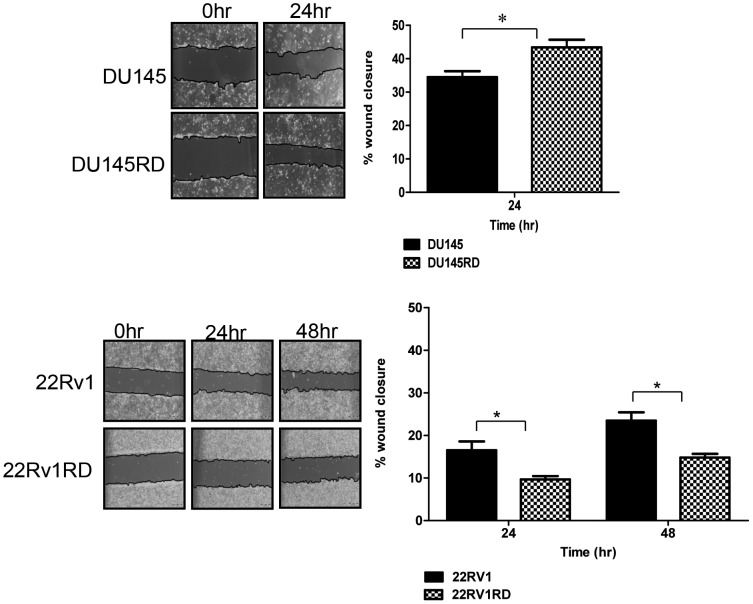
Cell motility. Wound-healing assays were performed to assess cell motility. Monolayers were scratched with a pipette tip and the resulting wounded areas were monitored by phase contrast microscopy for 24 hours (DU145) and 48 hours (22Rv1). Results are displayed as n = 3± SEM, where * = p<0.05 (Student’s t-test).

**Figure 3:**
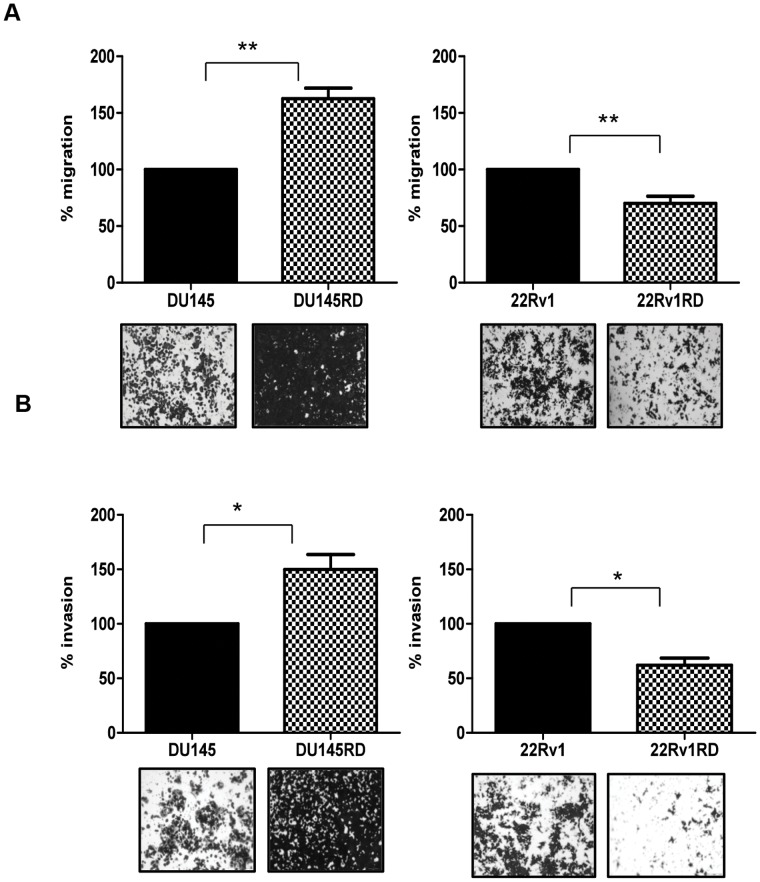
Cell migration and invasion. A: Migration assays were performed using 8 µm pore size 24-well transwell chambers; **B:** For invasion assays the inserts were pre-coated with ECM. Cells were allowed to migrate/invade for 48 hours (DU145) and 72 hours (22Rv1). Results are displayed as n = 3± SEM, where * = p<0.05, ** =  p<0.01 (Student’s t-test).

Observation of cell morphology did not reveal any substantial differences in the morphology of the cells following acquired docetaxel-resistance (Figure 1), although resistant cells proliferate more slowly than their docetaxel-sensitive parent populations (Table 2). Wound-healing assays were used to evaluate the effects of docetaxel-resistance on cell motility (Figure 2). After 24 hours, DU145RD cells demonstrated significantly (p<0.05) increased wound closure compared to DU145. Docetaxel-resistant 22Rv1RD showed marginal, but significant (p<0.05), decreased wound closure compared to 22Rv1. Considering cellular migration (Figure 3A) and invasion (Figure 3B), DU145RD was found to have increased motility (p<0.01) and invasive capabilities (p<0.05) when compared to DU145. 22Rv1RD compared to 22Rv1 cells, however, displayed a decreased trend in migration (p<0.01) and invasion (p<0.05).

**Table 2:** Average doubling time for prostate cancer cell lines and respective docetaxel-resistant variants.

Cell line variant	Doubling time (hr)
DU145	24.0±1.4
DU145RD	33.4±2.4
22Rv1	32.2±2.3
22Rv1RD	46.0±4.1

### Acquired Docetaxel-resistance Influences Anchorage-independent Survival and Growth

**Figure 4:**
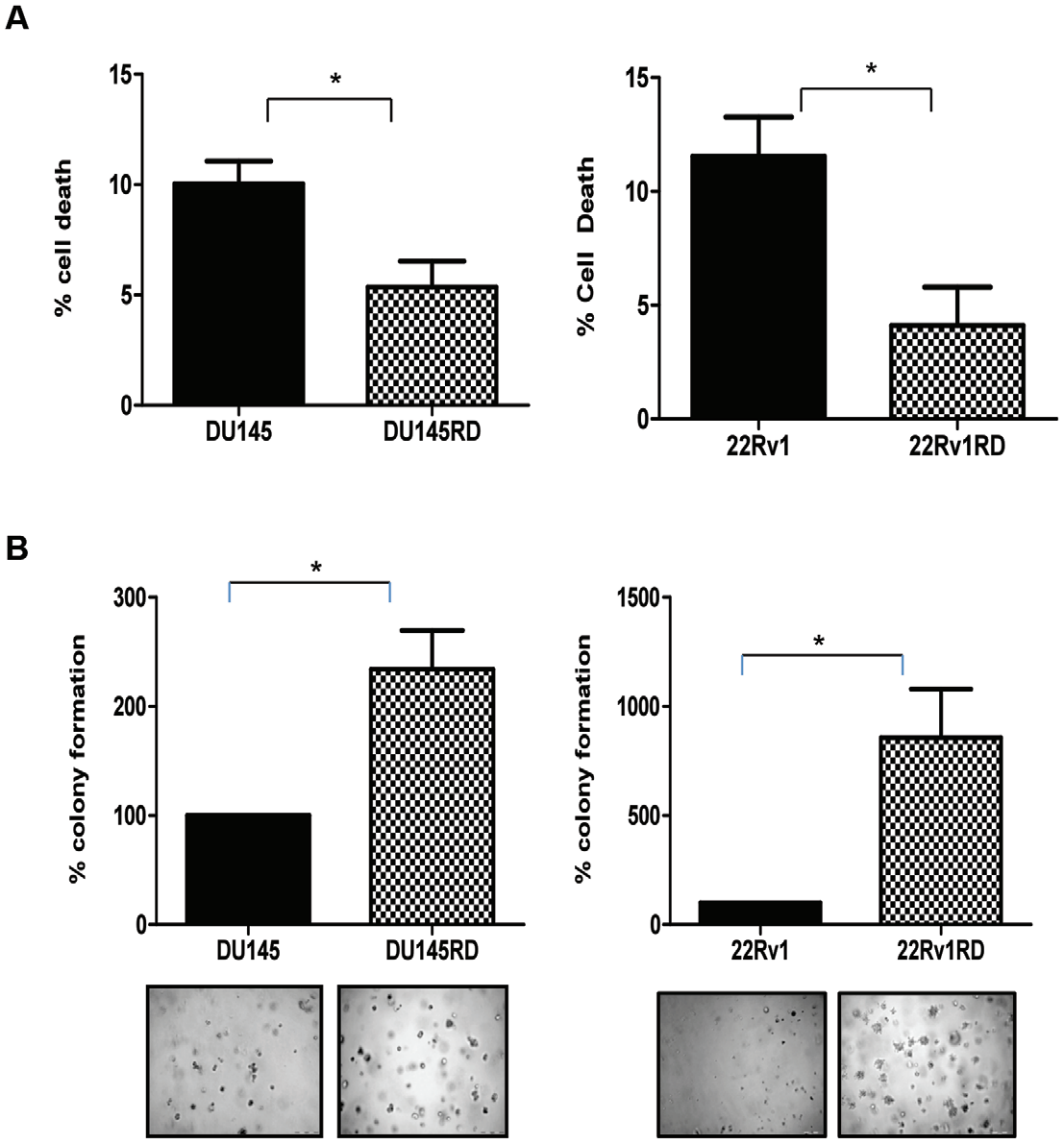
Anchorage independent growth. A: For *anoikis* assays, cell line variants were plated onto Poly (hydroxyethyl methactylic) acid-coated 24 well plates – or 95% ethanol-coated plates, as controls - and were cultured for 24 hours. 100 µl of Alamar blue dye was then added to each well, incubated for 4 hours and absorbance was measured at 570nm; **B:** Colony formation assays were performed using Cytoselect™ 96-Well Cell Transformation kit. Cells were incubated for 8 days in semisolid agar media before being lysed and detected with CyQuant GR Dye in a fluorescence plate reader. Results are displayed as n = 3± SEM, where * = p<0.05 (Student’s t-test).

Investigating if acquired drug resistance may also confer *anoikis* resistance, we observed drug resistance to be significantly associated with decreased cell death under *anoikis* conditions for both drug-resistant variants *i.e.* DU145RD (p<0.05) and 22Rv1RD (p<0.05); compared to their respective control cells (Figure 4A). Subsequently evaluating if these viable cells are also able to proliferate in suspension, colony formation in soft agar was found to be significantly increased in DU145RD (*P*<0.05) and 22Rv1RD (*P*<0.05) in comparison to their respective parent cells (Figure 4B).

### Exosomes are Secreted from Prostate Cancer Cells and can Influence Response to Docetaxel but do not Significantly Alter Proliferation, Motility and Invasion

**Figure 5:**
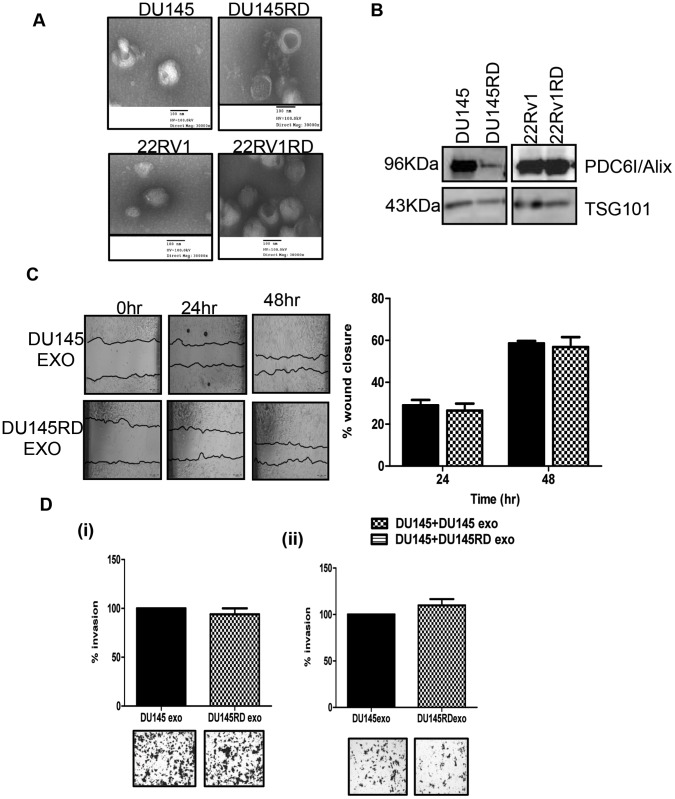
Exosome characterisation and assessment of affects on cell motility and invasion. **A:** Transmission Electron Microscopy was performed to investigate size and structure of exosomes; **B:** Western blotting was performed to assess the expression of common exosomes markers in 30 µg (TSG101) and 8 µg (PDC6I/Alix) exosomes isolated from DU145 and 22Rv1 cell line variants; **C:** DU145 wound-healing assays in the presence of exosomes (5 µg) from DU145 cell line variants; **D** (**i**)**:** DU145 invasion assays in the presence of exosomes (15 µg) from DU145 cell line variants; **D** (**ii**)**:** 22Rv1 invasion assays in the presence of exosomes (15 µg) from DU145 cell line variants.

Transmission electron microscopy identified the presence of exosomes isolated from the conditioned medium of all cell line variants (Figure 5A). Furthermore western blotting for both TSG101 and PDC6I/Alix (Figure 5B), proteins commonly associated with exosomes formation and thus considered to be important markers of successful isolation of exosomes [12], [13], were detected in isolates from the conditioned medium of all cell line variants. Amounts of exosomes expelled from the docetaxel-resistant and aged-matched variants did not differ significantly (Figure S1A). As acquired docetaxel-resistance in the DU145RD cells was associated with increased migration and invasiveness, we next investigated if autologous (DU145) or resistant (DU145RD) exosomes could alter motility assessed by wound-healing capacity (Figure 5C) and invasion (Figure 5D(i)) of DU145. No significant difference between wound closures of cells in the presence of DU145 or DU145RD exosomes was found. Similarly, when applied to 22Rv1 cells, neither DU145- nor DU145RD-derived exosomes conferred a substantial effect on invasion (Figure 5D(ii)).

**Figure 6:**
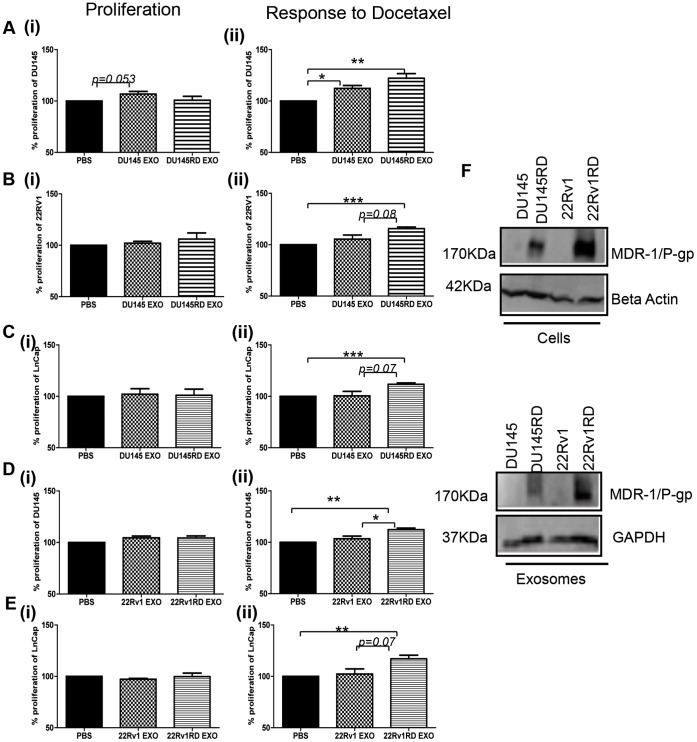
Isolation of exosomes and assessment of affects on toxicity assays. **A-C** (**i**)**:** DU145, 22Rv1 and LNCap proliferation in the presence of exosomes (20 µg) from DU145 cell line variants; **A–C** (**ii**) Response of DU145, 22Rv1 and LNCap cells to docetaxel in the presence of exosomes (20 µg) from DU145 cell line variants; **D–E** (**i**)**:** DU145 and LNCap proliferation in the presence of exosomes (20 µg) from 22Rv1 cell line variants; **D–E** (**ii**)**:** Response of DU145 and LNCap cells to docetaxel in the presence of exosomes (20 µg) from 22Rv1 cell line variants; **F:** Western blotting was performed to assess the expression of MDR-1/P-gp in total cellular protein (50 µg) and corresponding exosomes (30 µg) of DU145 and 22Rv1 cell line variants. Results are displayed as n = 3± SEM, where * = p<0.05, ** =  p<0.01, *** = p<0.001 (Student’s t-test).

A slight increase in proliferation of DU145 cells in the presence of DU145-derived exosomes was noted. This approached significance (p = 0.053); however, DU145RD exosomes did not significantly alter the proliferation of DU145 cells (Figure 6A(i)); possibly as a consequence of the slower growth rate of the cells from which these latter exosomes were derived. Following treatment with the IC_50_ concentration of docetaxel, the presence of DU145 exosomes (p<0.05) and to a greater extent DU145RD exosomes (p<0.01) (Figure 6A(ii)) were found to induce a significant level of docetaxel insensitivity (resistance) to the cells. To more broadly investigate if DU145RD exosomes can induce similar affects when applied to other cell lines, we next investigated these exosomes on both 22Rv1 and LNCap cells (Figure 6B and 6C). Neither DU145- nor DU145RD-derived exosomes significantly affected the proliferation of 22Rv1 or LNCap cells (Figure 6B(i) and 6C(i)). Following treatment with docetaxel, no substantial change in response of 22Rv1 or LNCap cells to docetaxel in the presence of DU145 exosomes was observed when compared to the affect of the drug in the presence of PBS instead of exosomes (Figure 6B(ii) and 6C(ii)). However, in the presence of DU145RD exosomes, the ability of 22Rv1 and LNCap cells to survive in this concentration of docetaxel was significantly increased (p<0.001) (Figure 6B(ii) and 6C(ii)). To determine if this is likely to be specific to exosomes from one cell line variant (DU145RD) or more general to exosomes from docetaxel-resistance cells, exosomes from 22Rv1 and 22Rv1RD variants were subsequently assessed. Here we found the same trend to occur *i.e.* there was no significant increase in proliferation of DU145 or LNCap cells in the presence of either 22Rv1 or 22Rv1RD exosomes (Figure 6D(i) and Figure 6E(i)). There was no significant change in response to docetaxel, by either DU145 or LNCap cells, in the presence of aged-parent 22Rv1-derived exosomes. However, in the presence of exosomes from the resistant variant 22Rv1RD, both DU145 cells (p<0.01) (Figure 6D(ii)) and LNCap cells (p<0.01) (Figure 6E(ii)) showed a significant increase in resistance to docetaxel.

We have previously reported that both DU145RD -and 22Rv1RD to a greater extent- expressed MDR-1/P-gp, whereas MDR-1/P-gp was undetected in the age-matched control cells, DU145 and 22Rv1 [14] (Figure 6F). To investigate whether MDR-1/P-gp is possibly carried *via* exosomes from DU145RD or 22Rv1RD cells, we investigated the presence of this efflux pump in exosomes isolated from these resistant variants. Here we report that MDR-1/P-gp is, in fact, present in the corresponding exosomal extractions (Figure 6F).

### Exosomes are Detected in Serum from Prostate Cancer Patients and can Influence Cellular Proliferation, Invasion and Response to Docetaxel

**Figure 7:**
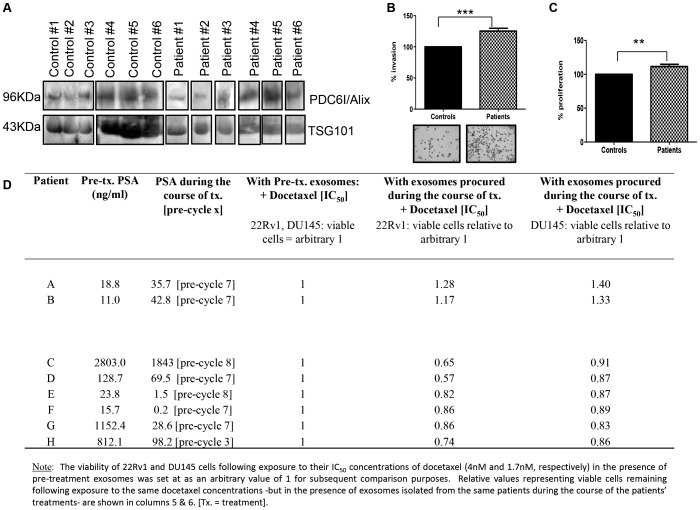
Isolation of exosomes from serum and assessment of affects on cells. A: Western blotting was performed to assess the expression of common exosomes markers in 30 µg exosomes isolated from sera of docetaxel-naïve patients (patient #1–6) and age-matched healthy controls (control #1–6). **B:** DU145 invasion in the presence of exosomes from docetaxel-naïve patients and aged-matched healthy controls (25 µg), with representative invasion image displayed. **C:** 22Rv1 proliferation in the presence of exosomes from treatment-naïve patients and age-matched healthy controls (25 µg). Results are displayed as n = 6± SEM, where ** = p<0.01, *** = p<0.001 (Student’s t-test). **D:** Response, to docetaxel, of 22Rv1 and DU145 cells in the presence of serum-derived exosomes from patients with elevated PSA levels (n = 2; Patients A & B) and in the presence of exosomes from patients with decreased PSA levels (n = 6; Patients C–H).

Western blotting for both TSG101 and PDC6I/Alix (Figure 7A) demonstrated the successful isolation of exosomes from sera of prostate cancer patients and healthy controls. Amounts of exosomes detected in the sera of prostate cancer patients and aged-matched controls did not differ significantly (Figure S1B). When added to DU145 cells, exosomes from docetaxel-naïve prostate cancer patients (n = 6) demonstrated a significant (*P*<0.001) increase in invasion when compared to exosomes from age-matched healthy controls (Figure 7B). Furthermore, when applied to 22Rv1 cells, there was a significant increase in proliferation in the presence of prostate cancer exosomes in comparison to those from healthy controls (*P*<0.01) (Figure 7C). For the purpose of assessing potential relevance of circulating exosomes with regards to how they may help predict or, indeed, affect patients’ response to docetaxel treatment, the influences of exosomes isolated from patients pre-docetaxel treatment (n = 8) compared to matched exosomes isolated from the same patients during the course of their 10 cycles of docetaxel treatment were assessed. For the purpose of this pilot study, patients (n = 6) who achieved decreasing PSA levels with treatment compared to their pre-treatment PSA levels were considered as “responders”, while those (n = 2) whose PSA levels increased during the course of treatment were considered as “non-responders”. As indicated in Figure 7D, 22Rv1 and DU145 cells were exposed to their approximate IC_50_ concentrations of docetaxel and the resulting cell viability was subsequently assigned an arbitrary value of 1. The exosomes isolated during the course of treatment from patients with increasing PSA levels (Patients A & B; “non-responders”) were found to protect both 22Rv1 and DU145 cells from the effects of docetaxel. In contrast, for the 6 docetaxel “responders”, the exosomes isolated during their course of treatment seemed to enhance the inhibitory effects of docetaxel on both the 22Rv1 and DU145 cells.

## Discussion

While docetaxel remains as the current gold standard for HRPC treatment, it only increases overall survival by on average 2.5 months and those patients who initially respond eventually develop resistance to this drug. In this study, we demonstrate the complex nature of docetaxel-resistance through the use of *in vitro* human prostate cancer cell line models. Both docetaxel-resistant cell line variants in this study demonstrated cross-resistance to doxorubicin, which is consistent with previous studies of docetaxel-resistant pancreatic cancer cells [15] and breast cancer cells [16]. In addition, paclitaxel, from which docetaxel is synthetically-derived, has been associated with cross-resistance to doxorubicin in paclitaxel-resistant prostate cancer cell lines [2]. Interestingly, DU145RD and 22Rv1RD cells, when compared to their aged-parent populations, also shared a number of other phenotypic characteristic changes in addition to resistance and cross-resistance. These included decreased proliferation rate, increased *anoikis* resistance and increased colony formation; but DU145RD and 22RV1RD differed with regards to changes in their motility and invasion.

Increased migration and invasion have previously been linked with chemoresistance [4]–[6] which are reflected in the findings of our DU145 model. While the 22Rv1 cell variants displayed the opposite effects with respect to migration and invasion, it is noteworthy that for the motility assays the level of wound closure was minimal following 48 hours and invasion/migration assays were seeded at a substantially higher density than required for DU145 cells to achieve the observed results. It is possible that these differences identified could be associated with already known differences between the cell lines (as detailed, 22Rv1 cells are from a primary tumour and are androgen-sensitive, while DU145 are from a brain metastasis and are androgen-insensitive). This further emphasises the need for these types of studies and the inclusion of larger panels of cell line models, where feasible, to as optimally as possible reflect other patient-to-patient diversities and so increase our understanding of the complexity of docetaxel-resistance in prostate cancer. The knockdown of beta catenin in osetocarcinoma cells increasing resistance to doxorubicin has previously been associated with suppressed invasion [7]. Furthermore, multi-drug resistant cell lines derived from the Dunning R3327 model of rat prostatic carcinoma have been shown to lose metastatic potential in host animals in comparison to their parent cells [17].

Previous studies using colorectal cancer (HT29), breast cancer (T47D) and colon cancer (H630) cells, in accordance with our own, have found that acquired chemo-resistance can reduce growth rate [18], [19]. Reduced cell proliferation due to the presence of chromosomal instability, which is associated with multi-drug resistance, has recently been demonstrated in colorectal cancer cells [20]. Thus the increased ability of docetaxel-resistant variants to resist cell death under *anoikis* conditions in our study cannot be attributed to a simple increased rate of proliferation. The association between increased clonogenic survival and chemo-resistance, observed in both our DU145RD and 22Rv1RD cells, is consistent with several other studies [18], [21], [22].

The emerging evidence that cells may communicate with neighbouring “or secondary cells” through the secretion of micro- or nano-sized vesicles (known as exosomes) carrying cellular information has recently highlighted the importance of these entities [23]. We, therefore, investigated the potential of these vesicles to be expelled and transfer phenotypic changes, associated with docetaxel-resistance, to secondary cells. No substantial differences in the motility, invasion or proliferation of DU145 and 22Rv1 cells were observed in the presence of DU145- or DU145RD-derived exosomes. Limited studies, to date, have demonstrated that exosomes expressing amphiregulin [24] and HSP90α [25] can increase cancer cell motility and migration. However, this does not appear to be the case with the prostate cancer cell-derived exosomes assessed here.

Interestingly we found induced docetaxel-resistance with DU145 cells in the presence of DU145RD exosomes (approx. 22%), compared to when these cells were exposed to their own exosomes. This suggests that DU145RD exosomes are, in fact, transferring resistance to docetaxel. Furthermore, a similar pattern was found when DU145- and DU145RD-derived exosomes were added to 22Rv1 and LNCap cells. Specifically, a significant increase in docetaxel-resistance (approx. 15% for 22Rv1; 16% for LNCap), independent of proliferation influences, resulted. This, for the first time suggests that exosomes may be a means of communicating docetaxel-resistance between cells. To confirm that the observed affects of DU145RD-derived exosomes is not limited to this cell line variant, we performed a similar investigation with 22Rv1 and 22Rv1RD exosomes. In keeping with the findings from our initial DU145RD exosomes assays, we observed a similar trend of conferred docetaxel-resistance (*i.e.* an increase of approx. 11–12%) to both DU145 and LNCap cells in the presence of 22Rv1RD exosomes.

MDR-1/P-gp is expressed by both our DU145RD and 22Rv1RD cells but is undetectable in the parent cells, implicating it as involved in the acquired docetaxel-resistance (this observation is somewhat in keeping with the clinical situation where the majority of prostate cancers are MDR-1/P-gp-positive [26]). Importantly, we found that the expression pattern in the corresponding exosomes reflected that of the cells from which they were derived, further supporting the potential of resistance transfer and our suggestion that MDR1/P-gp could potentially be -at least partly- involved in the newly-acquired resistance conferred by the exosomes. However, the difference in MDR-1/P-gp levels between exosomes from docetaxel-resistant variants compared to their parental cells does not necessarily imply a causal role for MDR1−/P-gp in the drug-resistance observed.

To further investigate the potential of exosomes in a clinical setting, as a pilot study we isolated exosomes from docetaxel-naïve prostate cancer patients and age-matched healthy controls. We investigated affects of these exosomes on cancer cell invasion and proliferation. The increased proliferation and invasion of cells in the presence of exosomes from cancer patients suggests a causative role for these exosomes. While this is a small pilot study, taken together with our cell line-derived exosomes studies, it further supports the hypothesis that exosomes may have a role in prostate cancer cell communication.

In the context of docetaxel-resistance, we subsequently investigated the relevance of exosomes derived from relevant patients. Specifically this pilot study (n = 8 patients) included matched serum exosomes obtained before and during the course docetaxel treatment. Here we assessed the response of DU145 and 22Rv1 cells to their IC_50_ concentrations of docetaxel (as shown in Table 1 *i.e.* 1.7 nM for DU145 cells; 4 nM for 22Rv1 cells) in the presence of these exosomes populations. The observed docetaxel-resistance conferred to both cell lines by exosomes isolated from pre-cycle 7 sera (n = 2 patients) correlated with the patients’ increase in PSA levels. The exosomes from the remaining 6 patients seemed to confer increased sensitivity to both DU145 and 22Rv1 cells, correlating with the patients (n = 6) decreased levels of PSA over their course of treatment.

A possible explanation for the manner whereby exosomes are affecting the phenotype of the “target” cells is that they are transferring mRNAs, miRNAs and/or proteins from the acquired resistance cells that are causal molecules in changing the cellular phenotype of the recipient secondary cells. This is in keeping with accumulating evidence that exosomes play an important role in cell-to-cell communication. For example, in 2007 Valadi et al. demonstrated that mRNAs carried via exosomes from mast cells are translated into protein on their transfer to target cells [10]. The successful uptake of exosomes by secondary cells was also demonstrated by Skog et al. [11] when fluorescently-labelled glioblastoma exosomes were incubated with endothelial cells. Many more recent examples of these observations have been reported and a recent opinion by Mittelbrunn and Sanchez-Madrid [27] further describes how this transfer of genetic information may occur, with the effects of exosomes on secondary cells potentially contributing to carcinogenesis and tumour growth; moulding the tumour microenvironment; promoting angiogenesis; and modulating immune response [28], [29]. Specifically in relation to modulating response to anti-cancer therapy, HER2-overexpressing exosomes from donor cells have been shown to decrease sensitivity of recipient cells to Trastuzumab [30]. So while we cannot speculate as to the particular molecules that may be carried *via* exosomes from our resistant cells to induce phenotypic changes in target cells (other than to suggest that the transfer of MDR-1/P-gp protein may, in part, be a contributing factor), it is reasonable to suggest that it may be a single -or combination of- mRNAs, miRNAs and/or proteins that have a causal role in docetaxel-resistance. Thus, now that functional relevance has been associated with these exosomes in conferring a level of docetaxel-resistance, profiling the content of these exosomes is warranted to better understand the precise molecule(s) and mechanism(s) involved.

In conclusion, this study corroborates that docetaxel-resistance in prostate cancer is highly-complex and may be associated with varied cellular affects in terms of resistance to other chemotherapeutic drugs, motility, invasion, and anchorage-independent growth. It is clear, however, that given the multi-faceted nature of docetaxel-resistance that not one, but several factors are likely to mediate their effects in prostate cancer. Here we show, for the first time, that cellular communication *via* exosomes may, in part, result in conferred docetaxel-resistance to secondary cells. Future studies on larger cohorts of serum specimens, from both docetaxel-naïve patients and also from patients following docetaxel treatment, are warranted –assessing both the affects of and the molecular contents of the exosomes– to expand our understanding of exosomes and their relevance to cell communication and docetaxel-resistance in prostate cancer.

## Materials and Methods

### Cell Lines and Cell Culture

Prostate cancer cell lines, 22Rv1 (ATCC CRL-2505; androgen-sensitive; from a primary human tumour), DU145 (ATCC HTB-81; androgen-insensitive; from a brain metastasis) and LNCap (ATCC CRL-1740; androgen-sensitive; from lymph node metastasis) were purchased from the American Type Culture Collection (ATCC). 22Rv1 and DU145 cells were maintained in RPMI medium (Sigma-Aldrich) supplemented with 10% fetal bovine serum (PAA), 1% L-Glutamine (Sigma-Aldrich) and at 37°C/5% CO_2_. LNCap cells were maintained in advanced RPMI (Biosciences) supplemented with 10% fetal bovine serum (PAA), 1% L-Glutamine (Sigma-Aldrich) and 1% Hepes (Sigma-Aldrich) and at 37°C/5% CO_2._ Docetaxel-resistant cell line variants, DU145RD and 22Rv1RD, were generated as previously described [14]. Age-matched parent cells (DU145; 22Rv1) were maintained in culture, unexposed to docetaxel, as controls for all experiments.

### Cytotoxicity Assays

Cytotoxicity assays for Docetaxel, Doxorubicin, 5-Fluorouracil and Carboplatin were performed on all age-matched parent cells and their docetaxel-resistant variants using the acid phosphatase assay. In brief, cells were seeded in 96 well plates at a density of 2×10^3^ cells/well (DU145 line variants) and 3×10^3^ cells/well (22Rv1 variants). The following day, cells were treated with a range of drug concentrations. Five days later, cell viability was evaluated as previously described [31].

### Cell Proliferation Assays

Cells were seeded 5×10^3^cells/well (in 24 well plates). Cells from 3 wells were trypsinised and separately counted at 24 hour, 48 hour, 72 hour, 96 hour, and 120 hour time-points. Doubling times were calculated using *Doubling Time Software v1.0.10* (http://www.doubling-time.com/compute.php).

### Wound-Healing Assays

Monolayers were scratched with a pipette tip and the resulting wounded areas were monitored, at indicated time-points, by phase contrast microscopy and determined using NIH Image J software [32].

### Migration and Invasion Assays

Assays were performed using 8 µm pore size 24-well transwell chambers (BD Sciences, UK). For invasion assays the inserts were pre-coated with ECM (Sigma-Aldrich). For the DU145 line variants, 5×10^4^cells/well (migration assays) or 1×10^5^ cells/well (invasion assays) were seeded in the upper compartment and allowed to migrate for 48 hours. For 22Rv1 variants, 6×10^5^ cells/well were seeded and allowed to migrate for 72 hours. Cells in the upper chamber were then mechanically removed and migrated cells were stained with crystal violet and photographed. Staining was solubilized in 10% acetic acid and absorbance was measured at 595 nm.

### Anchorage-independent Growth Assays

Assays were performed using Cytoselect™ 96-Well Cell Transformation kit (Cell Biolabs) according to the manufacturer’s instructions. For all variants, 7.5×10^3^ cells/well were incubated for 8 days.

### Anoikis Assays

DU145 and 22Rv1 cell line variants (1×10^5^ cells/well) were plated onto Poly(hydroxyethyl methactylic) acid-coated (Sigma-Aldrich) 24 well plates –or 95% ethanol-coated plates, as controls- and were cultured for 24 hours. 100 µl/well of Alamar blue dye (Sigma-Aldrich) was then added, incubated for 4 hours and assessed at 570 nm.

### Exosome Isolation from Conditioned Medium

For exosomes isolation, all cells were grown in RPMI medium supplemented with 5% of exosomes-depleted fetal bovine serum (dFBS) (PAA), 1% L-Glutamine (Sigma-Aldrich) and 1% penicillin/streptomycin (Invitrogen). FBS was depleted of exosomes by ultracentrifugation at 110,000 g for 16 hours [13]. Each cell line variant was seeded in nine 75 cm^2^ flasks at 2×10^5^ cells/flask (for DU145 variants) and 5×10^5^ cells/flask (for 22Rv1 variants). After allowing cells to attach over-night, medium was replaced and the cells were cultured for 3 (DU145 cell lines) or 5 (22Rv1 cell lines) days in the fresh medium; to approximately 80% confluency. Exosomes were subsequently isolated from conditioned medium (CM) using methods that we recently described [13]. The resulting exosomes pellets were resuspended in approximately 200 µl PBS and stored at −80°C for subsequent quantification (using BioRad protein assay Dye Reagent) and for analysis.

### Transmission Electron Microscopy

Exosomes were isolated from conditioned media as described above, and analysed by electron microscopy as previously described [33]. Briefly, approximately 10 µl of exosomes samples were placed on parafilm, in duplicate. A 300 mesh copper grid was placed on top of the drop and allowed to stand for 45 minutes. The copper mesh was subsequently washed thrice in fresh phosphate buffer for five minutes each, fixed in 3% glutaraldehyde for ten minutes, washed thrice for 5 minutes each in dH_2_O and contrasted in 2% uranyl acetate. Grids were then stored examined by electron microscopy at 100 kV using a JEOL JEM-2100 electron microscope.

### Exosome Isolation from Serum Specimens

Exosomes from serum specimens, procured through the All-Ireland Cooperative Oncology Research Group translational trial: 08–08, and with consent from the Ethics Committee of Beaumont Hospital, Dublin and the Mater Hospital, Dublin, were isolated using Exoquick (Systems Biosciences), as per manufacturer’s protocol. Serum from six prostate cancer patients (denoted by Patient #1–6) and age-matched healthy controls (Control #1–6) were used for invasion and proliferation assays. Exosomes isolated from further eight prostate cancer patients (denoted by Patient A-H) sera procured both before and during docetaxel treatment were used for “docetaxel-resistance”/cytotoxicity assays. For the purpose of these studies, patients who achieved decreasing PSA levels with treatment compared to PSA levels pre-treatment were considered as “responders”, whereas patients with elevated PSA following treatment were considered as “non-responders”.

### Western Blotting

Total cellular proteins were extracted from whole cells or exosomes using SDS lysis buffer (250 nM Tris-HCL, pH 7.4. 2.5% SDS). Protein quantification was performed using BioRad protein assay Dye Reagent (BioRad). Protein (50 µg for cellular protein samples and 30 µg for exosomes from CM samples for TSG101 and MDR-1/P-gp; 8 µg exosomes for PDC6I/Alix and 30 µg for serum exosomes) were separated on 7.5% SDS gels. Immunoblotting involved using the following primary antibodies: MDR-1/P-gp (Santa Cruz), PDC6I/Alix [34] (Abcam) and TSG101 [35], [36] (Abcam), GAPDH (Cell Signalling), β-actin (Sigma-Aldrich). Immobilon Western Chemiluminescent HRP substrate (Millipore) and a Bio-Rad ChemiDoc system were used to visualise the protein bands.

### Addition of Exosomes to Invasion, Migration, Proliferation and Cytotoxicity Assays

To assess any influences on cell motility using wound-healing assays, 5×10^4^ cells/chamber (DU145) were seeded in a 4-chamber slide and 5 µg exosomes from DU145 (autologous) or DU145RD (resistant) CM were applied/chamber, as appropriate. After 48 hours, monolayers were scratched and assessed as before.

To evaluate effects on invasion, invasion assays were seeded as detailed above, except that 3×10^5^ cells/insert were used for 22Rv1 cell line. DU145 or DU145RD exosomes (15 µg) or serum exosomes (25 µg) were applied as appropriate to each insert when cells had attached to the inside of the insert (∼ 4 hours). For proliferation and cytotoxicity assays, 4×10^3^ cells/well (DU145) and 5×10^3^ cells/well (22Rv1 and LNCap) was seeded in a 96 well plate and allowed attach over-night. For LNCap cells, plates were pre-coated with poly-d-lysine (Sigma-Aldrich) to facilitate adhesion. Equal quantities (20 µg) of cell-derived exosomes (DU145, DU145RD, 22Rv1, 22Rv1RD) or serum exosomes (25 µg) were then applied to each well, as appropriate. For cytotoxicity assays, the docetaxel IC_50_ concentration was applied to the wells, as set up for proliferation assay, at the same time as exosomes. As a control in proliferation and cytotoxicity assays, the equivalent volume of PBS was substituted where no exosomes were applied. Proliferation and cytotoxicity assays were incubated for 48 hours (for DU145 cells) or 72 hours (for 22Rv1 and LNCap cells) after which effects were assessed using the acid phosphatase method.

### Statistical Analysis

Statistical analysis was performed on Excel. *P*-values were generated using Student’s t-tests, with p<0.05 considered as statistically significant. Results are displayed as n = 3± standard error of mean. Except for clinical specimen proliferation and invasion studies which was n = 6± standard error of mean (patients versus controls). GraphPad was used for graph generation.

## Supporting Information

Figure S1 **Quantification of exosomes secreted by cells. A:** Following exosomes isolation, cells from flasks were trypsinised and counted (n = 3± SEM) and exosomes were quantified using BioRad protein assay Dye Reagent. Exosomes quantities were calculated per 10,000 cells; **B:** Exosome quantification per 200 µl of serum from treatment naïve patients and aged matched healthy controls (n = 6± SEM).
(TIF)
